# Protective effects of FCGR2A polymorphism in invasive pneumococcal diseases

**DOI:** 10.1186/cc11696

**Published:** 2012-11-14

**Authors:** A Bouglé, A Max, N Mongardon, D Grimaldi, F Pène, C Rousseau, JD Chiche, JP Bedos, E Vicaut, JP Mira

**Affiliations:** 1 Hôpital Cochin, Paris, France; 2 Institut Cochin, INSERM U1016/CNRS UMR8104, Paris, France; 3 Hôpital André Mignot, Le Chesnay, France; 4 Hôpital Lariboisière, Paris, France

## Background

*Streptococcus pneumoniae *is a major cause of pneumonia and meningitis. Several genetic polymorphisms have been described to explain differences in susceptibility and severity of encapsulated pathogen-related diseases. Among them, a functional *FCGR2A *polymorphism leading to amino acid change of histidine (H) to arginine (R) at position 131 appears to be a major candidate in adult invasive pneumococcal diseases (IPD). However, previous reports needed confirmation in a large well-defined population.

## Methods

A prospective genetic association study in a 24-bed medical ICU of a tertiary teaching hospital over 7 years. Patients were retrospectively selected from two prospective cohorts generated between January 2001 and December 2008. All Caucasian subjects with proven IPD and hospitalized in the ICU were included. Cases of IPD were defined by the isolation of *S. pneumoniae *from a normally sterile site (blood, bronchoalveolar lavage, quantitative tracheal aspiration, cerebrospinal fluid (CSF)). DNA from all Caucasian patients with IPD (pneumonia and/or meningitis) was genotyped for the FcγRIIa-R/H131polymorphism.

## Results

**Figure 1:**
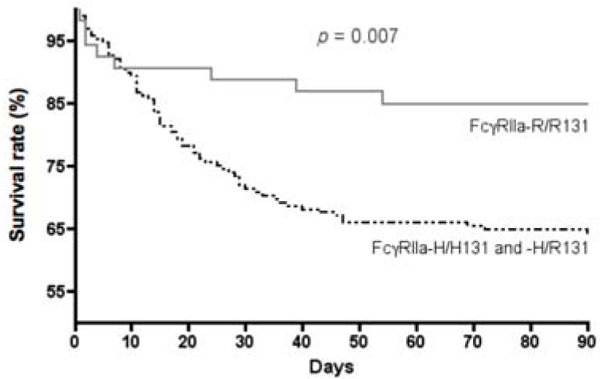
**Kaplan-Meier anlaysis of the probability of survival according to FcγRIIa polymorphism**.

Two hundred and forty-three patients with proven IPD were enrolled, 202 (82%) with pneumonia and 55 (22%) with meningitis. Mean age was 61 years, mean SAPS2 was 50.4, one-half of the patients had bacteremia, 84% of the cohort was mechanically ventilated and the hospital mortality rate was 31%. In the IPD group, distribution of the FcγRIIa-R/H131 genotypes (H/H: 25%; H/R: 53%; RR: 22%) was comparable with the distribution in the Caucasian control group. Comparison of the FcγRIIa-R/R131 and the (FcγRIIa-R/H131 + FcγRIIa-H/H131) groups did not demonstrate any difference for age, SAPS2, origin of sepsis and other co-morbid conditions. However, the variant FcγRIIa-R/R131 genotype was independently associated with decreased hospital mortality (OR = 0.251, CI 0.098 to 0.645; *P *= 0.004). See Figure 1.

## Conclusion

In a well-defined population of IPD patients, the frequency of the variant FcγRIIa-R131 does not differ from other critically ill patients. However, the FcγRIIa-R/R131 genotype was independently associated with increased survival regardless of the site of infection.

